# Development of a hollow fibre-based renal module for active transport studies

**DOI:** 10.1007/s10047-021-01260-w

**Published:** 2021-03-22

**Authors:** Alexandros Englezakis, Elnaz Gozalpour, Mohammed Kamran, Katherine Fenner, Elisa Mele, Karen Coopman

**Affiliations:** 1 Centre of Biological Engineering, Department of Chemical Engineering, Loughborough University, Loughborough, UK; 2 Clinical Pharmacology and Safety Sciences, R&D Biopharmaceuticals, AstraZeneca, Cambridge, UK; 3 Department of Materials, Loughborough University, Loughborough, UK

**Keywords:** Hollow fibre, 3D cell culture, Fluidic shear stress, Renal function, Drug transport

## Abstract

Understanding the active transport of substrates by the kidney in the renal proximal convoluted tubule is crucial for drug development and for studying kidney diseases. Currently, cell-based assays are applied for this this purpose, however, differences between assays and the body are common, indicating the importance of in vitro–in vivo discrepancies. Several studies have suggested that 3D cell cultures expose cells to a more physiological environments, thus, providing more accurate cell function results. To mimic the renal proximal tubule, we have developed a custom-made renal module (RM), containing a single polypropylene hollow fibre (Plasmaphan P1LX, 3M) that serves as a porous scaffold and compared to conventional Transwell cell-based bidirectional transport studies. In addition, a constant flow of media, exposed cells to a physiological shear stress of 0.2 dyne/cm^2^. MDCK-Mdr1a cells, overexpressing the rat Mdr1a (P-gp) transporter, were seeded onto the HF membrane surface coated with the basement membrane matrix Geltrex which facilitated cell adhesion and tight junction formation. Cells were then seeded into the HF lumen where attachment and tight junction formation were evaluated by fluorescence microscopy while epithelial barrier integrity under shear stress was shown to be achieved by day 7. qPCR results have shown significant changes in gene expression compared to cells grown on Transwells. Kidney injury marker such as KIM-1 and the hypoxia marker CA9 have been downregulated, while the CD133 (Prominin-1) microvilli marker has shown a fivefold upregulation. Furthermore, the renal transporter P-gp expression has been downregulated by 50%. Finally, bidirectional assays have shown that cells grown in the RM were able to reabsorb albumin with a higher efficiency compared to Transwell cell cultures while efflux of the P-gp-specific substrates Hoechst and Rhodamine 123 was decreased. These results further support the effect of the microenvironment and fluidic shear stress on cell function and gene expression. This can serve as the basis for the development of a microphysiological renal model for drug transport studies.

**Supplementary Information:**

The online version contains supplementary material available at 10.1007/s10047-021-01260-w.

## Introduction

A new drug is estimated to cost an average of $1.3 billion to develop over a 12-year period with half of that time being used for pre-clinical in vitro testing [1]. It involves several stages, models and techniques, each providing data for the Administration, Distribution, Metabolism, Elimination, Toxicity (ADMET) properties of New Chemical Entities (NCE). This balance between stringent tests and increasing costs make drug development highly complex and requires the combined effort of a multidisciplinary team over a long duration to complete. Current methods of drug screening incorporate mainly computational models, recombinant systems and in vitro 2D cell-based assays to predict the ADMET properties of NCE before proceeding to clinical trials.

Although these methods have revolutionised and industrialised drug development in terms of efficiency and speed of NCE screening, 90% of new drugs fail during clinical trials [2]. This indicates the discrepancy between in vitro and in vivo drug testing, and with current high drug development costs and attrition rates during pre-clinical testing, the pharmaceutical industry faces increasing investment risks and decrease in return of investment while drug development time is increased. Therefore, it is essential to improve and develop new screening models to reduce both time and cost, while producing compounds with more favourable ADMET properties for clinical trials.

Based on US’s Food and Drug Administration (FDA) regulatory guidelines, a new drug transport is suggested to study across the renal proximal tubule for better understanding the ADMET properties of a drug [3]. The renal convoluted proximal tubule is responsible for up to 80% of nutrient reabsorption/transport from the ultrafiltrate and drugs and their metabolites often accumulate in this region at high concentrations in intra- and intercellular spaces [4]. Thus, the proximal tubule is a major region of interest for studying the ADMET properties of NCEs and their interaction with important renal transporters belonging to the ATP-Binding Cassette (ABC) and Solute carrier (SLC) superfamilies [5–7].

A promising method for developing an accurate drug-screening model is the use of 3D renal cell cultures such as Organ-on-chips, organoids and bioartificial organs. Studies have shown that cells grown in a 3D environment using several methods, have characteristics closer to cells found in vivo, than their conventional 2D counterparts. These include cell growth, proliferation, phenotype, gene and protein expression profiles [8]. Unlike 2D cell cultures, cells grown in a 3D environment, provide a controllable environment in terms of pH, temperature, nutrient supply, waste removal and shear stress [9]. However, limitations such as the even distribution of oxygen and nutrients make their use complex.

Therefore, the design of a 3D cell culture system is crucial for its function and these range from the selection of scaffold/matrix, which is then seeded with the desired cells, or dispersing cells in a liquid matrix followed by polymerization [8].

Aebischer et al. have developed a hollow fibre (HF)-based bioreactor to be used as a bioartificial kidney (BAK) device [10]. These semi-permeable HF membranes were made from either an acrylic polymer or polysulphone and were seeded on the outer surface with two types of renal epithelial cell lines (MDCK or LLC-PK1). They have shown that cell confluence on the hollow fibres was reached within 3 weeks and ultrafiltration was maintained continuously without any anticoagulation. However, the morphology and function of these cells was altered depending on the HF material and cell loss was observed in the long term [10].

This concept is now used widely for BAK development for both clinical and pharmacodynamics applications. Humes et al. developed this design further with the use of an extracellular protein coating (pronectin-L) to enhance cell attachment, growth and polarisation [11]. The same group has scaled up this system to a multifibre bioreactor with cells grown on the outer surface of the HF and the extracellular spaces serve as the proximal tubule lumen for pro-urine formation while the inner HF served as the peritubular capillary [12]. They tested the BAK device on uremic animals and have shown that these cells replaced several metabolic processes lost in renal failure that could not be replaced with conventional haemodialysis. These included glutathione metabolism, vitamin D production and sufficient ammonia excretion [12]. They have also tested the same BAK configuration on patients in ICU suffering from acute renal failure. Patients have shown some improvement in regard to cardiovascular stability and renal function as determined by urine production. In addition, there was an inflammatory cytokine reduction (IL-6 and IL-10), therefore, the BAK had a protective effect from systemic inflammatory response syndrome (SIRS) and multi-organ failure. However, most patients exhibited low platelet production and hypotension, resulting in the termination of the clinical study [13, 14].

Although these HF-based BAKs are being developed mainly for renal replacement therapies and have seen significant progress, they still lack some basic proximal tubule functions that take part in excretory, metabolic and endocrine pathways [9]. This is due to several reasons, but most important is the type of cell used (cell line or primary), materials used, scalability and bioreactor design [15, 17]. In addition, the potential use of HF-based bioreactors for drug screening and renal transport studies has not been fully explored.

In this study, we have sought to develop a HF-based renal module (RM) similar to BAKs to be used as an in vitro renal transport model. A custom-made bioreactor was manufactured that houses a single Polypropylene HF (Plasmaphan P1LX, 3M).

It was hypothesised that due to their porosity and curvature, HFs are an ideal scaffold for renal proximal tubule epithelial cells, as it mimics the proximal convoluted tubule in the nephron, therefore, providing a similar 3D microenvironment. In addition, cells would be exposed to fluidic shear stress on both sides of the HF membrane to mimic the ultrafiltrate flow in the proximal tubule and blood flow in the peritubular capillaries. Renal MDCK-Mdr1a cells were seeded in the HF lumen, while syringe pumps provided a constant flow of media through the inner and outer HF membrane surface. Cells were then evaluated according to their tight cell monolayer formation, gene expression levels and renal transporter functionality compared to cells grown on Transwell inserts.

## Materials and methods

### Chemicals

Human collagen IV (0.3 mg/ml) and Valspodar were purchased from Sigma-Aldrich. DMEM (4.5 g/l glucose with l-glutamine, sodium pyruvate), Penicillin–Streptomycin (P/S, 100 U/ml), Foetal Bovine Serum (FBS), Fibronectin (from bovine plasma), rhLaminin521 (1 mg), Geltrex hESC-Qualified (Reduced Growth Factor Basement Membrane Matrix), Gibco TryplE, Rhodamine123, Hoechst 33,342, PBS (CaCl_2_, MgCl_2_ free, PFA (4%), anti-ZO-1 AF488 monoclonal antibody (1 mg/ml), AF-555 anti-rabbit antibody, BSA-AF594 and TRIzol were purchased from ThermoFisher Scientific. Plasmaphan P1LX HFs (0.45-μm pore size, 330-μm OD, 150-μm ID) were purchased by 3 M. MDCK-Mdr1a cell line overexpressing the rat P-gp was supplied by AstraZeneca. Anti-rat P-gp rabbit antibody was purchased from Abcam.

### Cell cultures

MDCK-Mdr1a cells were grown in DMEM (4.5 g/l glucose with l-glutamine, sodium pyruvate) media supplemented with 10% FBS and P/S (1 U/ml). Cells were expanded in conventional tissue culture plastic (TCP) and were always passaged when cells reached a confluency of 80%.

### HF coating and cell seeding on outer surface

P1LX HFs were cut into 3-cm segments, placed in 6-well ultra-low attachment plates, and held down to the bottom by stainless steel rings. A mixture of Ethanol/deionised water (70/30) was used to sterilise the HFs, followed by three washes with sterile water. Sterile HFs were then coated with either Human Collagen IV (200 μg/ml), rhLaminin521 (10 μg/ml), Fibronectin (10 μg/ml) or Geltrex (150 μg/ml) by submerging the HFs in the solutions and incubated 1 h at 37 °C. After coating, 4 × 10^5^ MDCK-Mdr1a cells were seeded and incubated for 3 days.

### SEM and fluorescence microscopy imaging

For SEM images, cells were fixed with 4% PFA (w/v), and after washing with PBS, they were left to dry at room temperature for at least 3 days. SEM images were taken using a Timochi, Benchtop SEM microscope (TM4000) and representative images are shown.

For fluorescence imaging after fixation, cells were stained with anti ZO-1 AF488 (2.5 μg/ml) conjugated antibody with or without anti P-gp (7 μg/ml) primary antibody overnight at 4 °C following with a 10-min DAPI (0.1 μg/ml) staining the next day. Samples with anti P-gp antibody were stained with AF-555 anti-rabbit antibody for 4 h at room temperature as well. After washing with PBS, samples were visualised with a Nikon, Eclipse Ti2 series fluorescent microscope.

For images showing the inner surface, cells were fixed and stained by injecting the prepared solution using a syringe inside the HF lumen. After staining and washing, the HFs were cut open diagonally or vertically with a scalpel to expose the inner surface.

### Renal module design and assembly

**Fig. 1:**
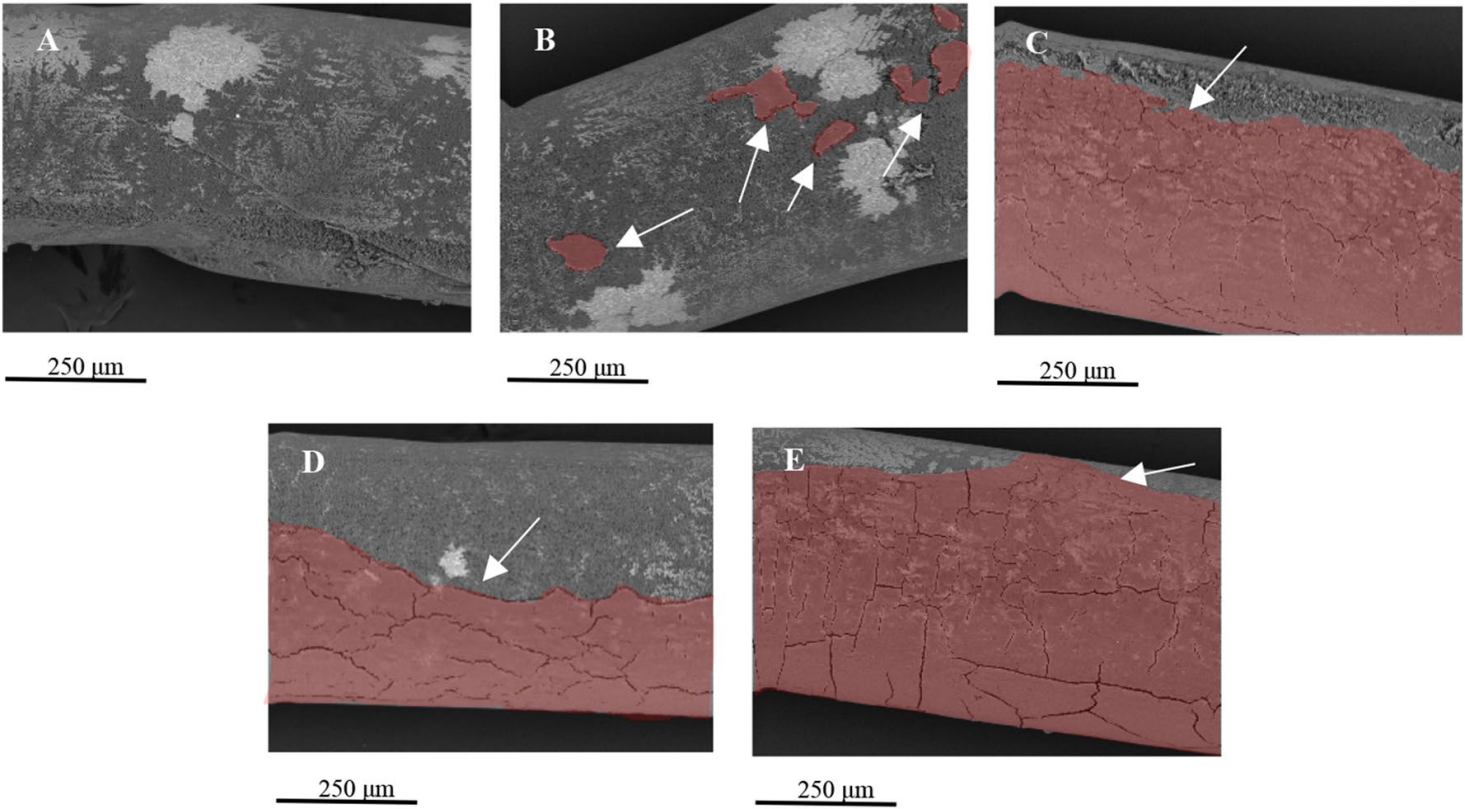
Representative SEM images of P1LX HFs seeded with MDCK-Mdr1a cells for 72 h on their outer surface. HFs were either uncoated (**a**) or coated with 200 μg/ml Human Collagen IV (**b**), 10 μg/ml rhLaminin521 (**c**), 10 μg/ml Fibronectin (**d**) or 150 μg/ml Geltrex (**e**). Cells are highlighted in red and shown by arrows

Four identical modules were designed to house a single 5-cm-long Plasmaphan P1LX HF with an extra capillary space (ECS) with a volume capacity of 0.25 ml while Luer lock stainless needles (30 G) were fed into the HF lumen (L). Independent inputs and outputs for both ECS and L were introduced into the modules and four-way stopcocks with male and female Luer locks were used to allow for module bypass to the waste bottle for air bubble removal and quick media change. Once assembled, the modules were connected to 10-ml BD Luer lock syringes while the flow was controlled by a syringe pump (Harvard PHD ULTRA™). PTFE tubing (Eppendorf DASGIP feed line) was used for connecting inputs/outputs from the feeding syringes to the collecting/waste bottles. Details on the BAK design and set up are shown in Fig. 1, Supporting information.

### Cell seeding

Once assembled, the modules were sterilised with 10 ml of 70% Ethanol with a flow rate of 100 μl/min, followed by a PBS wash of same volume and flow rate. HFs inside the modules were coated by injecting 1 ml of Geltrex in the lumen and incubate for 1 h at 37 °C. After coating, 1 ml of MDCK-Mdr1a cells (2 × 10^6^/ml) was injected into the lumen while the ECS was filled with DMEM complete cell culture media. After 24 h, unbound cells were flushed by applying a flow rate of 100 μl/min for 5 min. Afterwards, a shear stress of 0.2 dyne/cm^2^ was applied by perfusing media with a flow rate of 3 μl/min in both L and ECS. Cells grown in static conditions were seeded as mentioned above with daily media changes by applying a flow of 50 μl/min for 5 min. The flow rate was calculated using the following equation:

$$t = 4 \mu Q/\pi R^{3} ,$$

where *t* is shear stress (dyne.cm^2^), *μ* is media viscosity (Cp), *Q* is flow rate (cm^3^/s) and *R* is HF radius (cm).

### Cellular monolayer integrity assessment

To assess the tightness of monolayer in Transwell cell cultures, separate inserts were treated with Inulin-FITC (50 μg/ml) on the apical side and only plates with a *P*_app_ < 1 × 10^–6^ cm/s were considered to perform efflux assay.

In 3D cell culture, media flowing though the HF Lumen contained Inulin-FITC (50 μg/ml) and cell confluency was assessed daily by collecting sample from the ECS and measuring media fluorescence using a FLUOstar Omega plate reader at 485 nm ex–520 nm em. Once Inulin-FITC diffusion decreased and was stable for at least 3 days, it was assumed that cell confluency and monolayer formation was achieved in the HF lumen.

### qPCR

Cells grown under shear stress conditions were harvested and lysed in TRIzol. Total RNA was isolated, using the RNease Mini kit (Qiagen) followed by cDNA synthesis by High-Capacity RNA-to-cDNA™ kit (FisherScientific). Primers were designed and optimised for their specific targets (Table 1, Supporting Information). RT-PCR was performed with SYBR green master mix (ThermoFisher Scientific) and GAPDH was used as housekeeping gene, and relative expression levels were calculated as fold change from gene expression levels in 2D static cell culture (Transwells) using the 2^−ΔΔCT^ method. Statistically significant differences were determined by Student’s *t* test, where a *p* value of less than 0.05 was considered statistically significant.

### Lactate and glucose consumption

For Transwell cell cultures, 1 × 10^5^ MDCK-Mdr1a cells were seeded in a 24-well plate and daily samples were collected.

For RM cell cultures, cells were cultured as described above under flow conditions and samples were collected from the Lumen side of each module daily. Lactate and Glucose concentrations were measured using a Cedex Bio Analyzer (Roche Cedex Analyzer).

### Bidirectional transport assays

To study conventional Transwell cell culture bidirectional transport assays, 0.1 × 10^6^ MDCK-Mdr1a cells were seeded onto Corning Transwell inserts (12-well plate) and cultured for 10 days. The assessment of cellular monolayer and efflux assays of both Transwell and RM cell cultures were performed in parallel. The Transwell upper chamber is the Apical side while the lower chamber is the Basolateral side. The RMs HF Lumen (L) and Extracapillary space (ECS) were considered as the corresponding Apical and Basolateral sides, respectively.

Direction of substrate transport from the donor to the recipient side is shown with the symbols A > B (Apical to Basolateral) and B > A (Basolateral to Apical).

For Transwell BSA transport studies, after 10 days of culture, BSA-AF594 (50 μg/ml) diluted in DMEM media was added either in the Apical or Basolateral side of Transwell inserts and samples were collected from the receiver sides every hour.

For RM BSA transport, BSA-AF594 (50 μg/ml) diluted in DMEM was included in either the ECS (B > A) media or Lumen (B > A) media. Using a continuous flow rate of 3 μl/min, samples from the receiver sides were collected every hour.

For Transwell P-gp active transport assays, cells were grown for 10 days on Transwell inserts. On the final day, the inserts were washed with fresh media and incubated with 0.5 μM Valspodar or corresponding amount of DMSO for positive controls for 30 min. After that, media was prepared containing 20 μM Hoechst33342 or 10 μM Rhodamine123 with/without Valspodar (0.5 μM). Apical to Basolateral (A > B) transport was measured by adding the fluorescent substrate in the apical side and taking samples every 30 min from the basolateral side. For Basolateral to Apical (B > A) transport, the substrate was added on the basolateral side, and samples from the apical side were taken.

For RM P-gp active transport assays, MDCK-Mdr1a cells were seeded in the RM as described above. On day 9, cells were treated for 24 h with complete media containing the inhibitor Valspodar (0.5 μM) or equivalent amounts of DMSO. Afterwards, the ECS and Lumen sides were flushed completely and replaced with either DMEM with/without Valspodar (0.5 μΜ) or DMEM with/without Valspodar and fluorescent substrate (20 μM Hoechst or 10 μM Rhodamine123). Media perfusion was applied (3 μl/ml) and samples were collected every hour from the receiver sides.

Apparent permeability coefficients (*P*_app_) were calculated from the following equation:

$$P_{{{\text{app}}}} = \frac{Q/t}{{V\left( {C_{0} A} \right)}},$$

where *Q* is sample fluorescence (RFU), *dt* is time (s), *V* is receiver side volume (ml), *C*_0_ is donor fluorescence intensity (RFU) and *A* is insert membrane surface area (cm^2^). *P*_app_ ratios were calculated by calculating the ratio between *P*_app_ (B > A) and *P*_app_ (A > B). For cellular monolayer assessment, separate inserts were treated with Inulin-FITC (50 μg/ml) on the apical side and only plates with a *P*_app_ < 1 × 10^–6^ cm/s were considered. Hoechst fluorescence was measured at 355 nm ex/520 nm em while Rhodamine123 was measured at 485 nm ex/520 nm em.

Statistically significant differences were determined by Student’s *t* test, where a *p* value of less than 0.05 was considered statistically significant.

## Results

### HF coating

**Fig. 2:**
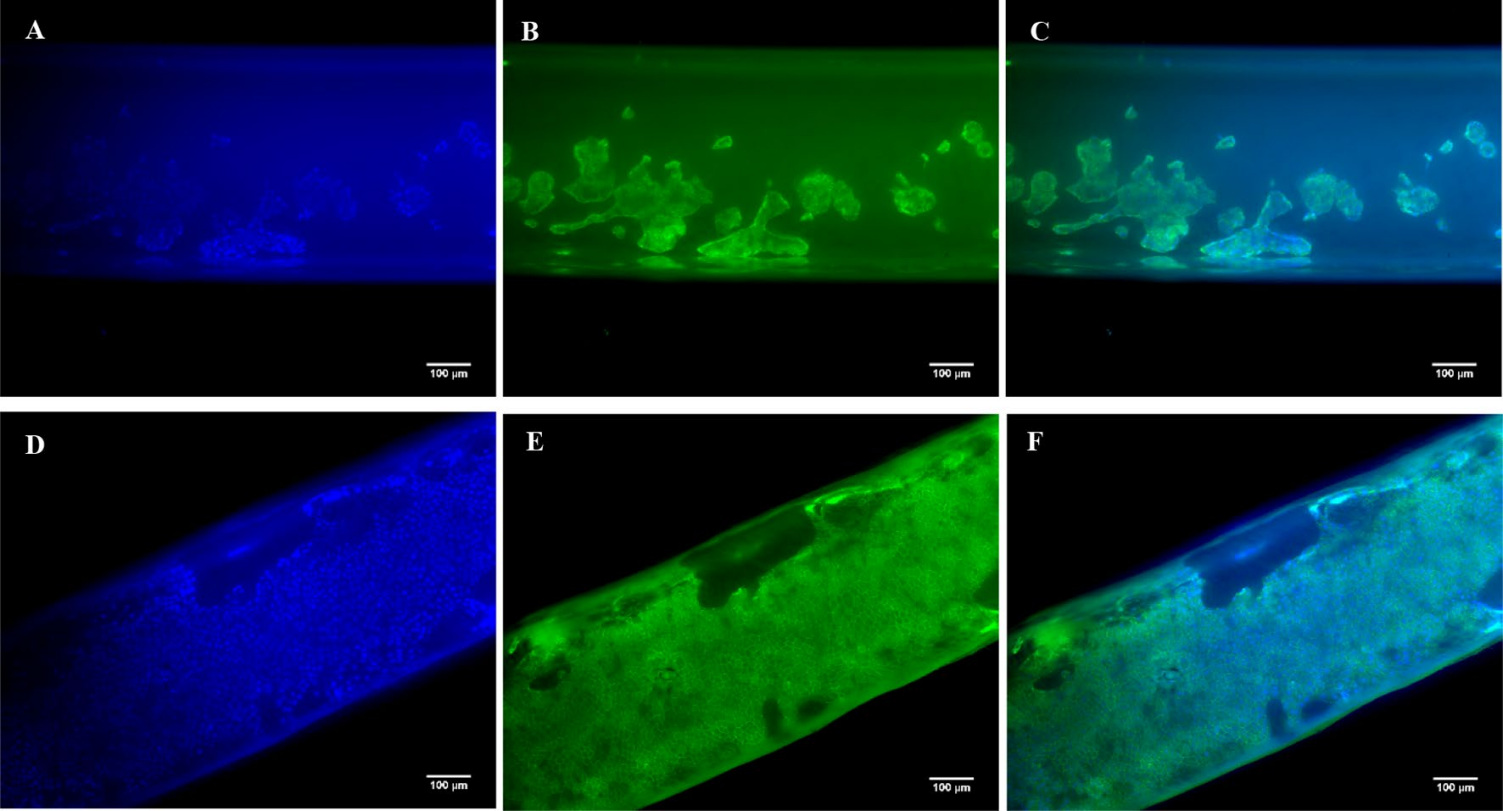
Representative fluorescent microscopy images of MDCK-Mdr1a seeded for 72 h on the outer surface of uncoated (**a**–**c**) and coated (**d**–**f**) P1LX HF membrane. Cells are shown stained with DAPI (**a**, **d**), anti ZO-1 AF488 (**b**, **e**). Merge of both channels are also shown (**c**, **f**)

First, a coating with a suitable basement membrane matrix protein was examined. As the aim of this initial study was for cells to attach in sufficient numbers on the HF membrane, cells were seeded on outer surface only. Comparison of MDCK-Mdr1a attachment on coated P1LX HFs using different basement matrix proteins is shown in Fig. 1. Cell adhesion and HF coverage is shown to be greatly affected on the type of basement matrix protein used. Greater cell HF coverage is seen by HFs coated with Geltrex, Laminin and Fibronectin. Limited cell coverage is seen when human collagen IV coating was used and almost no cells were seen on uncoated HFs. Fluorescent staining of P1LX HFs has shown greater cell densities on Geltrex-coated HFs (Fig. 2). Furthermore, ZO-1 staining indicates tight junction formation on cells attached on HFs.

### Effect of shear stress on MDCK-Mdr1a cells

**Fig. 3:**
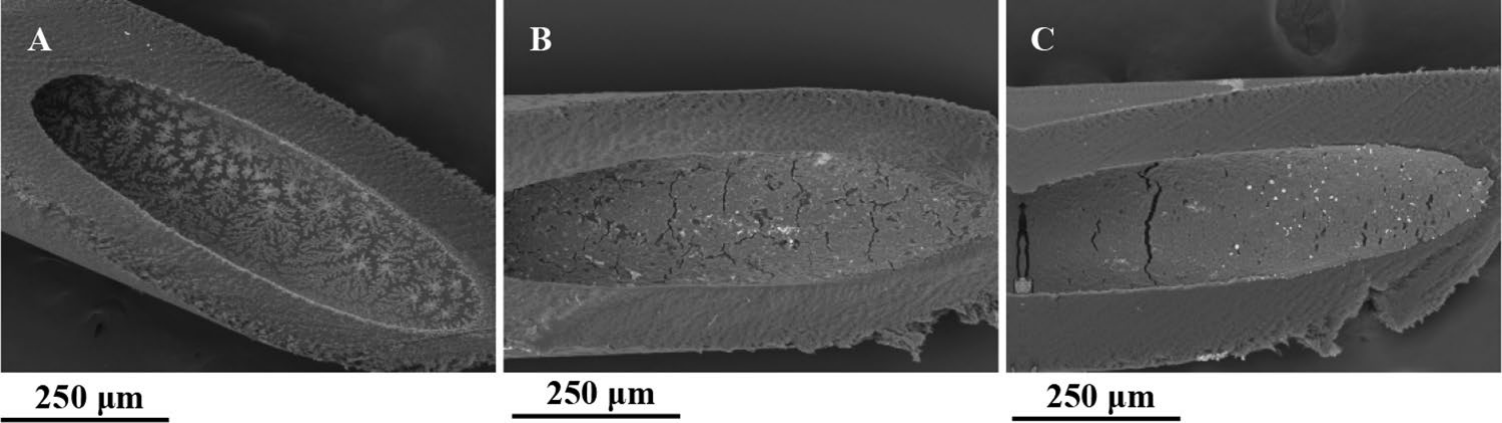
Representative SEM images of HF inner surfaces without cells (**a**) and MDCK-Mdr1a cells, cultured in the renal module under static (**b**) and shear stress conditions (**c**)

We next sought to examine whether MDCK-Mdr1a cells can withstand constant shear stress due to media perfusion though the RM. Cells were seeded in the HF lumen of the RM and were exposed to a constant flow of media after 24 h to induce shear stress. Figure 3 shows SEM images of open P1LX HFs with cells grown in static or flow conditions. Cells grown in static conditions show gaps and inconsistent cell monolayer (Fig. 3b). In contrast, cells exposed to shear stress seem have complete HF surface coverage with minimal gaps (Fig. 3c).

**Fig. 4:**
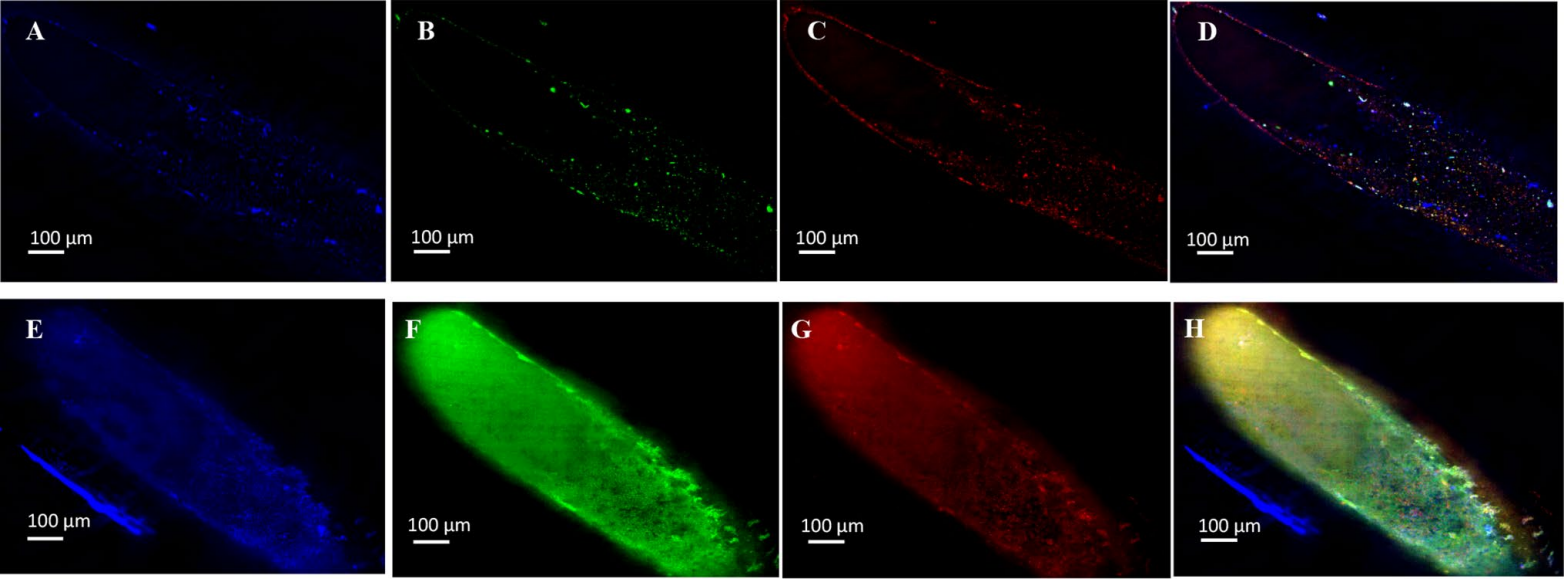
Representative fluorescent microscope images of HF inner surfaces with MDCK-Mdr1a cells grown in coated HFs under static (**a**–**d**) and fluid shear stress (**e**–**i**) conditions. Cell nuclei were stained with DAPI (**a**, **e**), and ZO-1 (**b**, **f**) and P-gp (**c**, **g**) were stained with their corresponding antibodies and channels were merged (**d**, **g**)

**Fig. 5:**
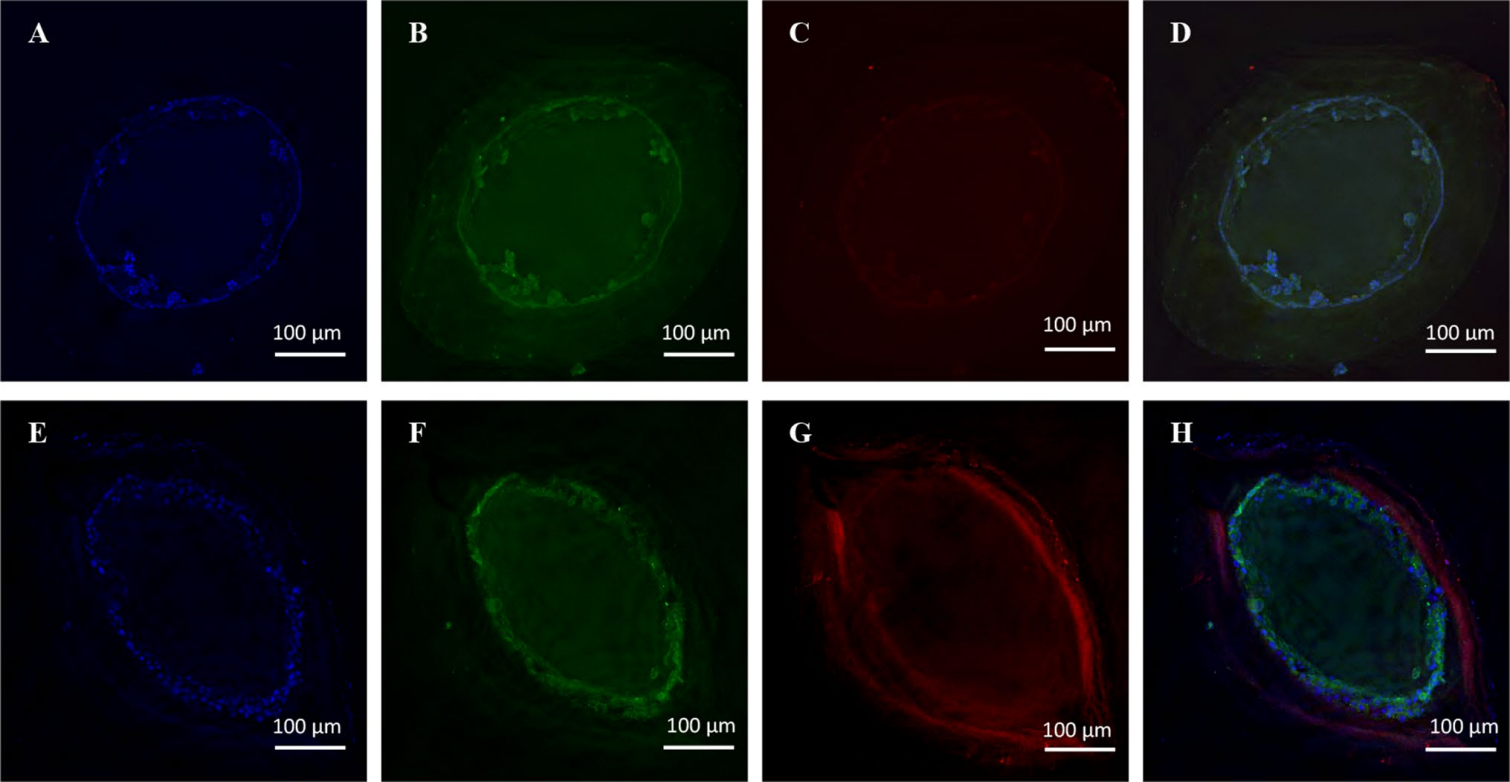
Representative fluorescent microscope cross-section images of HFs seeded with MDCK-Mdr1a cells grown under static (**a**–**d**) and fluid shear stress (**e**–**i**) conditions. Cell nuclei were stained with DAPI (**a**, **e**), and ZO-1 (**b**, **f**) and P-gp (**c**, **g**) were stained with their corresponding antibodies and channels were merged (**d**, **g**)

This is further supported by fluorescence microscopy images that show MDCK-Mdr1a cells cultured in flow conditions, grew in higher densities, and formed clear tight junctions due to staining of ZO-1 marker compared to cells grown in static conditions (Fig. 4). P-gp expression is seen in both conditions (Fig. 4c, g). Furthermore, HF cross-sections show differences in cell growth between the two conditions. Statically grown cells form clusters towards the lumen centre, while shear stress induces the formation of a cell monolayer on the HF inner surface (Fig. 5).

### Cellular monolayer integrity in renal modules

**Fig. 6:**
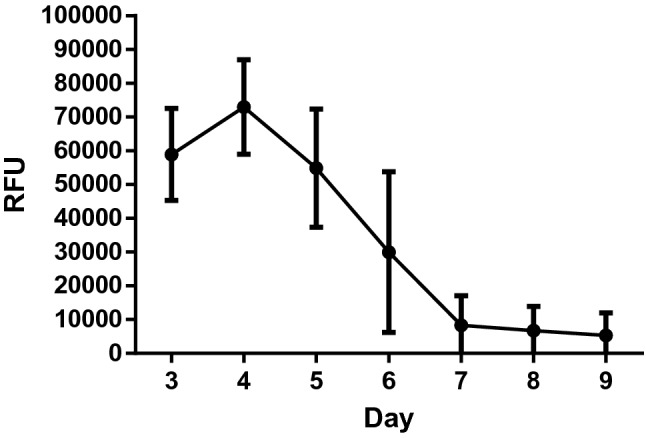
Daily diffusion of Inulin-FITC from HF lumen to the outer ECS chamber in the RM (mean value ± SD, *n* = 3)

Cell confluency and tight junction formation were assessed by the diffusion of the fluorescent marker Inulin-FITC from the HF lumen to the ECS. Inulin-FITC diffusion to the ECS is decreased and reached minimal levels by day 7 and maintained this until day 9 indicating the presence of an intact cellular barrier (Fig. 6).

### qPCR

**Fig. 7:**
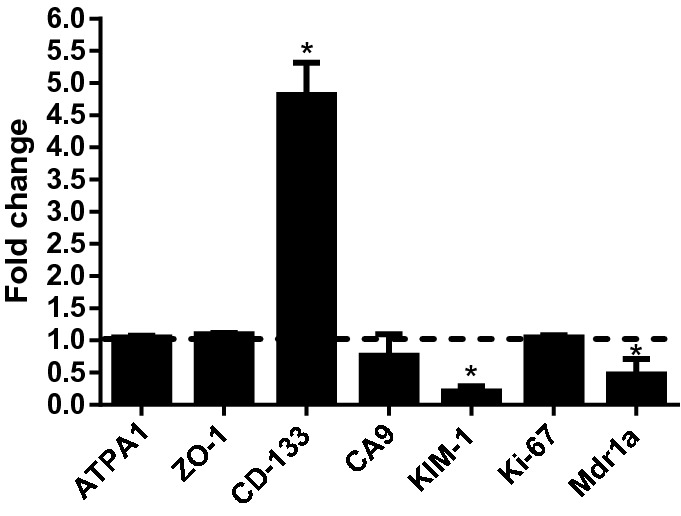
Gene expression fold change in MDCK-Mdr1a cells seeded in the RMs under shear stress. Gene expression levels were normalised to GAPDH housekeeping gene and fold change was calculated to gene expression levels of cells grown on Transwell inserts. Dotted line indicates the normalised value of “1” for 2D cell cultures’ gene expression profile. *p < 0.05

Later, we studied the changes in gene expression between polarised MDCK-Mdr1a cells grown on Transwells and cells grown in the RMs under flow conditions (Fig. 7). CD133 (Prominin-1) has shown a fivefold increase compared to cells grown in 2D, while the kidney injury molecule KIM-1 and the renal transporter P-gp (Mdr1a) have shown a downregulation of 80% and 50%, respectively. Na^+^/K^+^ ATPase (ATPA1), tight junction protein (ZO-1), carbonic anhydrase 9 (CA9) and proliferation marker (Ki-67) have shown no significant changes.

### Glucose and lactate consumption (Transwell vs RM)

**Fig. 8:**
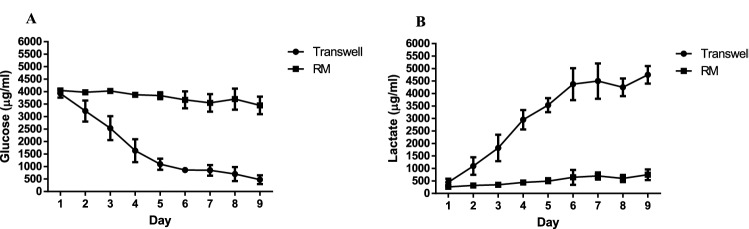
Representative data of daily glucose concentration (**a**) and lactate concentration (**b**) of cells grown in the RM under flow conditions and compared to static Transwell cell cultures (mean value ± SD, *n* = 2)

Glucose consumption and Lactate accumulation were measured daily in MDCK-Mdr1a cell cultures grown in the RMs and compared to conventional Transwell cell cultures (Fig. 8). Transwell cell cultures have shown a steady decrease and increase of Glucose and Lactate, respectively, while RM cell cultures maintained constant concentrations under flow conditions.

### Bidirectional transport assays (Transwell vs RM)

**Fig. 9:**
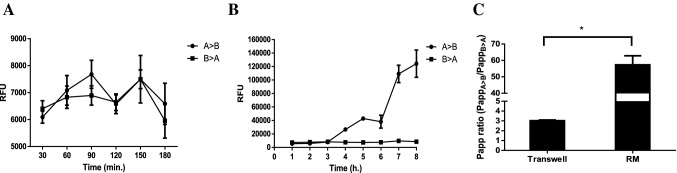
Representative data of BSA-AF594 transport in MDCK-Mdr1a cells grown on Transwell inserts (**a**) and in RM cell cultures (**b**). Permeability coefficient (*P*_app_) ratios were calculated from 2 separate experiments, *p* values calculated using a paired *t* test were *p < 0.05 (**c**) (mean value ± SD, *n* = 2)

The ability of cells grown in a 3D environment in the RMs under flow conditions to transport specific substrates was studied and compared to static Transwell cell cultures. BSA reabsorption and transport from the apical to the basolateral sides was shown to increase steadily (Fig. 9a, b) in both conditions, while permeability coefficient ratios of 3D cell cultures have shown a 20-fold increase in BSA reabsorption compared to 2D Transwell cell culture models (Fig. 9c).

**Fig. 10:**
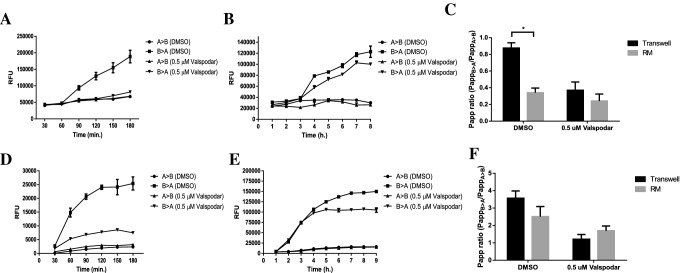
Representative data of efflux of the P-gp-specific substrates Hoechst (**a**–**c**) and Rho123 (**d**–**f**) and a comparison between Transwell efflux (**a**, **d**) and RM efflux (**b**, **e**). Permeability coefficient (*P*_app_) ratio comparison between the two models are also shown (**c**, **f**) and statistical significance was calculated using a paired *t* test where *p < 0.05 (mean value ± SD, *n* = 3)

We next sought to study the function of the apical transporter P-gp and compare its Hoechst and Rho123 transport activity in Transwell and RM cell cultures. Clear efflux of both Hoechst and Rho123 from the basolateral side (ECS) to the apical side (L) is seen in the RMs while inhibition with Valspodar confirms the P-gp mediated transport (Fig. 10b, e). Comparison with Transwell cell culture assays show a reduction of *P*_app_ ratios in RMs, indicating a decrease in P-gp function. Significant reduction is seen in Hoechst efflux, while Rho123 transport was affected slightly compared to Transwell-based assays (Fig. 10c, f).

## Discussion

In this study, we sought to develop a HF-based RM to be used as a more physiological model for bidirectional transport. The HF of choice was Plasmaphan P1LX, 3M. This polypropylene-based HF membrane is inherently hydrophobic, and thus, unfavourable for cell adhesion. However, Buschmann et al*.* have managed to develop an artificial artery model by seeding endothelial cells on fibronectin-coated P1LX HFs suggesting the necessity of basement membrane proteins in supporting cell attachment [18].

Thus, we initially tested several coating methods that could support MDCK-Mdr1a cell attachment on the HF membrane’s outer surface. Human collagen IV, Fibronectin and rhLaminin521 are some of the most common coatings while Geltrex was also examined [16, 19–22]. Geltrex is a mixture of laminin, collagen IV, entactin and heparin sulphate proteoglycans, proteins found in the human proximal tubule basement membrane matrix in vivo [23]. Therefore, it was hypothesised that Geltrex could provide more physiological conditions for renal cell attachment.

The greatest membrane coverage by attached cells was seen by Geltrex-coated HFs, while collagen IV-coated HFs had the lowest. In addition, no cells have attached on the uncoated HFs, indicating the importance of coating methods for cell adhesion promotion. Due to its pre-diluted condition by the manufacturer, Geltrex is stable for longer periods at room temperature, and gelation can be prevented. Gelation is a common issue with several basement membrane matrices during coating, which could cause blockages in the HF lumen and disturb media perfusion inside the RM [24, 25]. Furthermore, MDCK-Mdr1a cells were able to form tight junctions on Geltrex-coated HFs as seen by the staining of ZO-1. Tight junctions are crucial for both cell–cell interactions and tight monolayer formation [26–28]. Cell interactions are important for maintaining tissue structure and function while a tight cell monolayer allows for substrate active transport controlled by epithelial cell transporters rather than diffusion [29].

We next sought to study the effects of media perfusion through the RM and exposure of MDCK-Mdr1a cells to shear stress. Fluid shear stress is essential for cell morphology microvilli formation and function [30–32]. Renal proximal tubule epithelial cells are under constant fluid shear stress and are able to adapt to blood and ultrafiltration flow fluctuations [33]. Furthermore, 3D renal models with other cell lines and bioreactors have suggested a connection between shear stress and renal transport gene expression [34, 35]. The exact mechanism that causes this is still unknown.

We have shown that when exposed to shear stress, cells were grown in greater numbers and have had an almost complete coverage of the HF lumen. In addition, when exposed to shear stress, MDCK-Mdr1a cells formed tight junctions and a monolayer while under static conditions, cells formed clusters growing towards the HF lumen centre, while staining of the ZO-1 marker decreased. P-gp staining has shown expression of Mdr1a in cells grown in either static or flow conditions.

For qPCR studies, we have examined seven markers that are involved in renal cell function. ATPA1 expresses for Na^+^/K^+^ ATPase, an important ion pump found in a wide variety of cell types including kidney epithelial cells and play an important role in regulating signal transduction pathways [36]. ZO-1 is highly expressed in cells forming tight junctions while CD133 is present in cells that have plasma membrane protrusions such as microvilli [37, 38]. CA9 is a widely used endogenous marker for hypoxic cells and KIM-1 is a kidney injury marker expressed in epithelial cells under stress or epithelial cell damage [39, 40]. Finally, proliferation was observed by measuring Ki-67 expression and P-gp function by Mdr1a expression.

qPCR results have shown a fivefold upregulation of the microvilli marker CD133 in comparison to static Transwell cell cultures. This suggests an increase in cell membrane protrusion activity, and it may involve increased microvilli length and function. Furthermore, KIM-1, a marker of acute kidney injury, is downregulated by ~ 80%, indicating that cells are not under stress or damaged [40]. In addition, CA9 was slightly downregulated, meaning cells are not under hypoxic conditions in the RM. Furthermore, ATPA1 and Ki-67 have remained unchanged, indicating cells have a comparable metabolism and proliferation rates as cells grown in 2D. The P-gp encoding gene Mdr1a, however, was downregulated by ~ 50% indicating significant changes in key renal transporters under shear stress. Changes in P-gp expression in cells exposed to shear stress has been reported in other studies as well. Jang et al. and Vriend et al. have reported an increase in P-gp expression in primary human proximal tubule epithelial cells using microfluidic cell culture systems [41, 42]. However, Jayagopal et al. have reported a decrease in P-gp expression in MDCK cells when shear stress was introduced [35].

Following the establishment of MDCK-Mdr1a cells’ ability to grow under flow conditions, we have shown that Glucose and Lactate concentrations remain constant within the RM, compared to static Transwell cell cultures. This indicates the importance of a constant flow system compared to static cultures where the cycle of nutrient/metabolic waste accumulation/depletion causes significant changes to cell function [43].

The next step was to evaluate the MDCK-Mdr1a cells’ ability to form an intact cell barrier. The formation of a tight cell monolayer in the HF surface is crucial for the model as this would allow the transfer of a compound through the renal cell active transport system rather than diffusion across a porous membrane. Due to the non-transparency of both the HF and RMs, an alternative approach was implemented. Media flowing through the HF lumen contained Inulin-FITC and the leakage to the ECS was measured daily. As cells cover the inner walls of the HF and form tight junctions, less Inulin-FITC is leaked and by day 10, almost none passes through indicating the presence of an intact cell barrier.

Active renal reabsorption and tubular secretion are crucial functions of the proximal tubule in the nephron, and it is initiated mainly by transporters located on the apical and basolateral sides of proximal tubule epithelial cells. Renal protein reabsorption is one of the key features of renal physiology where more than 60% of the absorption takes place at the apical membrane of cells in the proximal convoluted tubule [44]. Albumin is the most abundant protein in the plasma and it although a portion filters through the glomerulus, most is reabsorbed through receptor-mediated endocytosis in the proximal convoluted tubule and then into the bloodstream [45].

Unidirectional flux of fluorescently labelled BSA (BSA-AF594) of cells grown in the RMs indicates that cells not only retain one of the most important functions of the proximal convoluted tubule, but they do so in greater efficiency than static Transwell cell cultures. The almost 30-fold increase in the *P*_app_ ratio show large differences in albumin reabsorption between the two models.

Finally, we have studied P-gp function. As an ATP-binding cassette transporter located at apical membrane, it mediates the efflux of exogenous and endogenous compounds including potential therapeutics by secreting them into the tubular lumen [46, 47]. Hoechst and Rho123 are known P-gp-specific fluorescent substrates while Valspodar inhibits its action [48, 49]. Efflux assays have shown significant and minor differences in P-gp-mediated efflux between Transwell and RM cell cultures depending on the substrate. The reduction in substrate efflux *P*_app_ ratios could be explained by several factors such the downregulation of P-gp expression described earlier or the slower diffusion of substrates across the HF. The Transwell membrane is 10 μm thick, while the HF membrane is 150 μM thick which could hinder substrate diffusion [50].

In conclusion, we have developed a custom-made HF-based bioreactor (RM) to be used for studying renal transport studies. Differences between Transwell and RM cell cultures were shown, making it a candidate as a 3D renal model. However, several factors could contribute to these changes other than shear stress and scaffold curvature. Oxygen concentration, cell interactions with the HF surface and pore size have not been properly addressed. In addition, the RM has not been compared to the active transport of renal cells in vivo to conclude if it induces cells grown in vitro to have a more physiological phenotype.

Further studies using pharmaceutical compounds and primary renal cells would be required to determine the possibility of using the RM as a drug-screening tool, while co-cultures with endothelial cells in the ECS could further enhance the imitation of the in vivo physiology.

## Supplementary Information

Below is the link to the electronic supplementary material.

Supplementary file1 (DOCX 336 KB)
