# Supplementary material for: Development of a hollow fibre-based renal module for active transport studies
Source: J Artif Organs. 2021 Mar 22;24(4):473–84. doi: 10.1007/s10047-021-01260-w (PMC8571221; doi:10.1007/s10047-021-01260-w)
Supplement: Supplementary file 1 — Supplementary file1 (DOCX 336 KB) [file 10047_2021_1260_MOESM1_ESM.docx]

1. **Supporting information**

| **Table 1 (Oligonucleotide primer sequences for qPCR)** | | | | |
| --- | --- | --- | --- | --- |
| **Gene** | **Function** | **Species** | **Forward Primer (5’-3’)** | **Reverse Primer (5’-3’)** |
| GADPH | Housekeeping gene | Canis lupus | aacatcatccctgcttccac | gaccacctggtcctcagtgt |
| ATPA1 | Na^+^/K^+^ ATPase | Canis lupus | tcagagtggtgtctcgttcg | gcacagctcgatgcatttta |
| ZO-1 | Tight junction marker | Canis lupus | cggtaccagctcctctcttg | cggtttggtggtctgaaagt |
| CD-133 | Microvilli marker | Canis lupus | ttgtctgctgtttgctgacc | ctgattccaactccgaccat |
| CA9 | Hypoxia marker | Canis lupus | taagcagctccacaccctct | ggactggctcaaaagacctg |
| KIM-1 | Kidney injury molecule | Canis lupus | gtggagtcacaaccatgtgc | caacacggcaacaatactgg |
| Ki-67 | Proliferation marker | Canis lupus | ctgcttgtttggaaggggta | gcacaggctcatcaatagca |
| Mdr1a | P-gp renal transporter | Rattus norvegicus | cgttgcctacatccaggttt | tggagacgtcatctgtgagc |


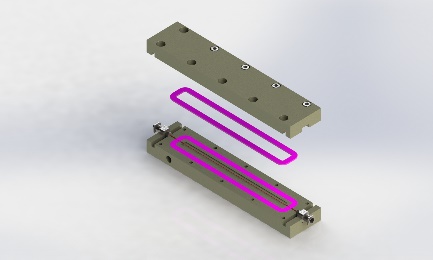

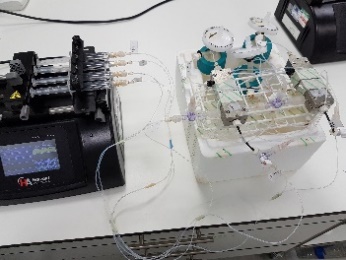

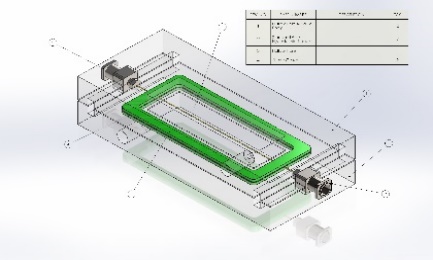


**A**

**B**

**C**

**ELS Bypass (to waste)**

**LUMEN Collection**

**ELS Collection**

**Stainless steel needle (30G)**

**LUMEN Bypass (to waste)**

**4-way stopcock**

**4-way stopcock**

**Stainless steel needle (30G)**

**Module**

**O ring**

**ELS chamber**

**P1LX HF**

**ELS INPUT**

**LUMEN INPUT**

Supp. Fig.1: 3D design of renal modules (A,B) and photo of a complete set up with syringe pump (C). A schematic of a renal module with a HF in the ECS chamber and media flow directions (D).

**D**
